# Psychometric evaluation of the respiratory syncytial virus infection, intensity and impact questionnaire (RSV-iiiQ) in adults

**DOI:** 10.1186/s12955-023-02174-2

**Published:** 2024-02-20

**Authors:** Valerie Williams, Carla DeMuro Romano, Lyn Finelli, Shanshan Qin, Todd L. Saretsky, Jia Ma, Sandy Lewis, Matthew Phillips, Richard H. Osborne, Josephine M. Norquist

**Affiliations:** 1 RTI Health Solutions, Box 12194, 3040 East Cornwallis Road Post Office, Research Triangle Park, NC 27709-2194 USA; 2 Merck & Co., Inc., Rahway, NJ USA; 3 Measured Solutions for Health P/L, Victoria, Australia

**Keywords:** Respiratory syncytial virus (RSV), RSV infection, intensity and impact questionnaire (RSV-iiiQ), Measurement properties, Symptoms, Impact, Disease burden, Psychometric evaluation

## Abstract

**Background:**

Despite a number of respiratory syncytial virus (RSV) vaccine candidates being tested in clinical trials, disease-specific, self-reported instruments assessing symptom severity of RSV infection from the perspective of adult patients are still needed. The RSV Infection, Intensity and Impact Questionnaire (RSV-iiiQ) was adapted from the Influenza Intensity and Impact Questionnaire (FluiiQ™). This study evaluated some measurement properties of the RSV-iiiQ.

**Methods:**

Data were collected in a web-based survey over two consecutive days. Participants completed the RSV-iiiQ, the Patient Global Impression of Severity, Sheehan Disability Scale, Patient Global Impression of Change, EQ-5D-5L, and a demographic questionnaire. Test-retest reliability, internal consistency, construct validity, and responsiveness of the RSV-iiiQ scales were assessed.

**Results:**

111 adults with RSV were enrolled and self-reported a variety of symptoms across the range of disease severity via a web-based platform. The RSV-iiiQ scales demonstrated satisfactory test-retest reliability, construct validity, and discriminating ability. One-factor confirmatory factor analyses confirmed that each of the four scales was sufficiently unidimensional, and internal consistencies indicated that the computation of RSV-iiiQ scale scores was plausible. Correlation-based analyses provided support for the construct validity of the RSV-iiiQ scores, and known groups analyses supported discriminating ability. Estimates of responsiveness of the scale scores were also satisfactory.

**Conclusions:**

RSV infection is highly symptomatic and causes significant disease burden, and self-report instruments assessing symptom severity and impact are important for evaluation of new treatments. This study describes the preliminary psychometric properties of the RSV-iiiQ and indicates this tool may be useful for the assessment of the severity of symptoms and impact of acute RSV infection in adults. The findings also indicated two items, Runny nose and Ear pain, may be unnecessary and should be revisited using item response theory analysis with a larger sample size.

**Supplementary Information:**

The online version contains supplementary material available at 10.1186/s12955-023-02174-2.

## Background

Respiratory syncytial virus (RSV) causes upper and lower respiratory infections and can lead to severe disease primarily in young infants and older adults (≥ 65 years of age) [1, 2]. Although awareness of respiratory diseases caused by RSV is high among pediatricians, awareness is lower among health care providers who care for adults. In adults, there are an estimated 5 million medically attended cases [3], 177,000 hospitalizations [4], and 11,000–17,000 deaths annually in the United States (US) [4–6]. The annual incidence of RSV infection is estimated to be 5.5% in community-dwelling older adults [4] and 5-10% in older adults living in congregate settings [7]. The winter season incidence rate among older adults can reach nearly double that of influenza depending on a range of factors, including seasonal factors and the availability of influenza vaccination [4]. Similar to influenza, RSV mortality disproportionately affects the elderly, with 78-82% of all RSV-associated deaths occurring in persons ≥ 65 years of age in the US [5, 6]. An estimated 1-2% of all cardiorespiratory deaths are attributed to RSV [5, 6].

Despite the considerable burden of RSV in the community and to the health care system, there are no licensed vaccines, and only symptomatic treatment is available. However, there are a growing number of RSV vaccine candidates under development [8]. An instrument is needed that can evaluate the ability of RSV vaccines to reduce the severity and duration of symptoms (e.g., cough, malaise, shortness of breath) and the time to return to usual activities of daily living. Patient-reported outcome (PRO) instruments assessing symptom duration and severity can be used to measure these endpoints in clinical trials, but first PRO instruments must be rigorously developed and evaluated to assure that they can generate valid and reliable data.

We modified an existing PRO instrument, the Influenza Intensity and Impact Questionnaire (FluiiQ™) [9], to develop the Respiratory Syncytial Virus Infection, Intensity and Impact Questionnaire (RSV-iiiQ) to assess the symptoms and impact of RSV infection from the perspective of adults with acute RSV. As described in our companion paper [10], in-depth concept elicitation and cognitive debriefing interviews with adults who had RSV provided evidence for the relevance of the proposed RSV-iiiQ items. In this study, we assessed the preliminary psychometric properties of the RSV-iiiQ consistent with standards described in US Food and Drug Administration (FDA) guidance [11, 12], including its measurement structure, internal consistency, test-retest reliability, construct validity, responsiveness, and discriminating ability.

## Methods

### Study Design

This psychometric evaluation study used a web-based, noninterventional, observational design that received approval from the RTI International Institutional Review Board (Federal-Wide Assurance #3331).

### Participants

The study was conducted in a convenience sample of US participants recruited by two large university medical clinics located in Ann Arbor, Michigan, and New York, New York, and two research facilities headquartered in Philadelphia, Pennsylvania, and Raleigh, North Carolina. Traditional single power/sample size calculations are not easily applied to psychometric evaluation studies due to the variety of methods used. A sample size of 30 per subgroup provides approximately 0.80 power to detect standardized effect sizes (i.e., Cohen’s d) of 0.50 [13] and correlation coefficients of 0.50 [14]. A sample size of 100 provides a 90% confidence interval half-width of 0.1 based on an expected test-retest intraclass correlation coefficient (ICC) of at least 0.7 [15]. Therefore, we chose 100 as the target sample size.

Inclusion criteria consisted of participants having (1) experienced symptoms indicative of RSV infection within 21 days of a physician diagnosis or healthcare visit involving RSV, (2) had a polymerase chain reaction–confirmed positive test for RSV within the last 21 days, and (3) provided written informed consent for participation. Individuals were not eligible to participate if they (1) presented with a comorbid respiratory condition with symptoms similar to RSV, (2) were currently receiving supplemental oxygen therapy for chronic lung disease or heart disease, (3) had received a diagnosis of any oncologic disease treated with chemotherapy in the previous 12 months, or (4) had participated in an investigational medicinal product study during the previous 30 days.

### Outcome Instruments

The psychometric analyses focused on the RSV-iiiQ [10], a PRO measure of symptoms and impact of RSV infection adapted from the FluiiQ™ [9] in alignment with FDA guidance [12]. The use and adaptation of the FluiiQ^™^ was authorized under license from Measured Solutions for Health P/L, Australia. The RSV-iiiQ includes 29 questions in four hypothesized scales: Respiratory Symptoms, Systemic Symptoms, Functional Impacts, and Emotional Impacts [10]. All RSV-iiiQ questions use a 4-point response scale (0 [“None” or “No difficulty”] to 3 [“Severe” or “Severe difficulty”]), and scale scores are computed as the average of corresponding items, with higher scores indicating worse symptoms or impacts. The recall period is “the past 24 hours.” Data were collected over 2 consecutive days. On day 1, participants completed the RSV-iiiQ, a Patient Global Impression of Severity (PGIS) rating, the Sheehan Disability Scale (SDS), the EQ-5D (EQ-5D-5L), and a demographics questionnaire (e.g., gender, race, age). On day 2, participants completed the RSV-iiiQ, a PGIS, a Patient Global Impression of Change (PGIC), and the EQ-5D-5L. The PGIS/PGIC, SDS, and the EQ-5D-5L are widely used generic PRO instruments and were selected as additional measures to assist in the validation and interpretation of the RSV-iiiQ data.

On day 2, the PGIC [16, 17] asked participants to provide an overall assessment of recent change in the severity of their RSV symptoms on a 5-point scale (“Since I first began the study, my RSV symptoms are now: Much better (1); A little better (2); No change (3); A little worse (4); Much worse (5)”). The PGIS asked participants to rate their overall RSV symptom severity on a 4-point scale (1 = None; 2 = Mild; 3 = Moderate; 4 = Severe).

The SDS [18] is a widely used, self-rated questionnaire designed to measure the extent to which an individual’s disability due to an illness or health problem interferes with work/school, social life/leisure activities, and family life/home responsibilities over the past week. The SDS uses a numeric rating scale from 0 (Not at all) to 10 (Extremely) and also includes questions assessing the number of school days or workdays lost and the number of days that were underproductive due to symptoms.

The EQ-5D-5L [19, 20] is a generic health-related quality of life measure that provides a brief and simple measure of current health status (i.e., “today”). Participants rated five domains—Mobility, Usual activities, Self-care, Pain/discomfort, and Anxiety/depression—using five ordered response categories. In addition, the EQ-5D-5L includes a 20-centimeter visual analog scale (VAS) on which respondents rate their global health state. The EQ-VAS is scored from 0 to 100 (worst to best health imaginable). The EQ-5D-5L profile/domain scores were used in this study, without utility scores.

### Statistical analysis

Analyses, as described in detail below (Table 1), were aligned with FDA guidance documents for PRO instruments and clinical outcome assessments [12, 21, 22]. Analyses were conducted using all participants who completed the day 1 assessment. In addition to effect sizes and statistical significance, patterns of results were emphasized. Mplus version 7.4 [23] was used for confirmatory factor analyses using item-level polychoric correlation matrix with mean- and variance-adjusted weighted least-squares estimation with a probit link. Other analyses were conducted using SAS version 9.4 or higher [24].

**Table 1:** Definitions and Criteria for Psychometric Measurement

Property and Definition	Test and Criteria
**Distribution of scores** Standard descriptive statistics to characterize average scores and variability and identify unanticipated response anomalies	Means (and medians, modes) and standard deviations (and score minimums and maximums) should be within acceptable ranges; patterns of scores should be as expected Frequencies of answers to each question should not be extremely skewed, i.e., many “best” or “worst” scores
**Structure** The relationships among questions and the extent to which they belong together for scoring purposes	Inter-item correlations should be positive, ranging from approximately 0.30 to 0.80
	Item-total correlations should be positive and ≥ 0.30
	Internal consistency/Cronbach’s alphas between 0.70 and 0.95 [33]
	Factor analysis model fit Factor loadings ≥ 0.30 Comparative fit index (CFI) > 0.95 [34, 35] Standardized root mean square residual (SRMR) < 0.06 Root mean square error of approximation (RMSEA) < 0.05 [28, 36] Tucker-Lewis Index (TLI) > 0.95 [35, 37]
**Test-retest reliability** Stability of scores over time when no change is expected in the concept of interest	For categorical scores, kappa coefficients ≥ 0.21 indicate fair agreement [38] For continuous scores, intraclass correlation coefficients > 0.70 [25, 39]
**Known groups validity** The degree to which scores can distinguish among known groups hypothesized a priori to be different	Scores should be able to distinguish among groups hypothesized to be different [21], for example, scores should be statistically better among groups of patients with less severe disease
**Construct validity** Evidence that relationships among scores conform to a priori hypotheses regarding logical relationships that should exist with other measures or characteristics of patients	The extent to which observed correlations among measures match hypothesized correlations in terms of sign and magnitude. Criteria for acceptability depend on the degree of conceptual similarity between the scores of interest and other instruments. A moderate (r = 0.30 to 0.49) or strong (r ≥ 0.50) correlation [40] is considered evidence of convergent construct validity; small (r = 0.10 to 0.29) or trivial (r < 0.10) correlations do not generally provide evidence of construct validity
**Responsiveness** Evidence that scores are capable of detecting change	Effect size (ES) estimates (calculated as: [change from day 1 to day 2] ÷ [day 1 SD]) and standardized response means (SRMs) show change over time Large (ES or SRM ≥ 0.80), moderate (ES or SRM = approximately 0.50), small (ES or SRM ≤ 0.20) [40] Observed score changes should be statistically different from 0, tested with paired *t* tests

### Distribution and internal structure

Response frequency distributions for each RSV-iiiQ item were tabulated at day 1 and day 2 to examine potential floor and ceiling effects. Inter-item correlations and item-total correlations for the RSV-iiiQ questions were computed to examine whether the patterns of relationships supported the measurement structure, expected scoring, and validity of the RSV-iiiQ data collected [25].

The RSV-iiiQ is based on the established FluiiQ™ [9]; the modified conceptual framework and general item groupings of RSV-iiiQ were endorsed by patients in previous qualitative research [10]. Because of the evidence from the FluiiQ™ and the qualitative results, the expected users of the RSV-iiiQ, and the potential label claim strategy, we considered only subscale scores to be of interest, rather than total score. Therefore, a priori single-factor confirmatory factor analysis (CFA) models for each set of questions were undertaken: Respiratory Symptoms (9 questions), Systemic Symptoms (8 questions), Functional Impacts (8 questions), and Emotional Impacts (4 questions).

### Reliability

Because it was assumed that minimal change in patients’ conditions would occur between 2 consecutive days, the test-retest stability of the RSV-iiiQ was assessed using day 1 and day 2 RSV-iiiQ question and scale scores. Because different symptoms can worsen and improve rapidly over the course of RSV infection, the test-retest analysis used only those participants who reported “no change” on the PGIC at day 2 to ensure that variability was not due to changes in RSV. Kappa coefficients were computed to assess the test-retest reliability of the RSV-iiiQ questions [15]. For scale scores, ICCs were computed using a two-way (subjects × time) mixed-effects analysis of variance (ANOVA) with absolute agreement for single measures [26]. To estimate the internal consistency of each RSV-iiiQ scale score, Cronbach’s coefficient alpha [27] was computed.

### Concurrent and known groups validity

Pearson’s and polyserial correlation coefficients (*r*) were computed to examine the construct validity of the RSV-iiiQ scores with the goal of testing whether stronger relationships exist between instruments assessing similar constructs as compared with instruments assessing more disparate constructs. Correlations between RSV-iiiQ scores and the SDS were expected to be positive and moderate (r = 0.30 to 0.49) to strong (r ≥ 0.50), and it was specifically hypothesized that the Functional Impacts score would correlate relatively strongly with the SDS Work/School and the SDS Family Life/Home Responsibilities scores. Moderate positive correlations were expected between the RSV-iiiQ and EQ-5D-5L domain scores, and it was hypothesized that the Emotional Impacts score would correlate more strongly with the EQ-5D-5L Anxiety/depression domain than with the other EQ-5D-5L domains and that the Functional Impacts score would correlate more strongly with the EQ-5D-5L Usual activities domain and the EQ-5D-5L Self-care domain.

Known groups analyses compared various subgroups of interest to provide evidence regarding the discriminating ability of the RSV-iiiQ. For example, analyses of variance (ANOVAs) examined mean differences in RSV-iiiQ scores between participants classified according to number of days since RSV diagnosis (≤ 7 days vs. ≥ 14 days), global ratings (PGIS = Moderate or Severe vs. PGIS = None or Mild), and SDS scores (SDS Work/School > 5 vs. ≤ 5; SDS Family Life/Home Responsibilities > 5 vs. ≤ 5). It was hypothesized that RSV-iiiQ mean scores indicating worse symptoms would be observed among participants more recently diagnosed (≤ 7 days), reporting worse scores on the SDS (SDS Work/School > 5; SDS Family Life/Home Responsibilities > 5), and with greater overall RSV symptom severity as reported in the PGIS (PGIS = Moderate or Severe).

### Responsiveness

The RSV-iiiQ’s ability to detect change (i.e., responsiveness) when change in symptoms and impact is expected was evaluated using effect-size estimates of change and paired t tests that compared the differences between day 1 and day 2 scores.

## Results

A total of 111 eligible adults with RSV were screened, recruited, and completed the day 1 web-based survey; of these participants, 95 (85.6%) also completed the day 2 web-based survey. Table 2 presents the participant characteristics and the baseline descriptive statistics for additional outcomes of interest. Similar RSV symptoms were reported on both day 1 and day 2 by participants (Additional File 1, Table S1). The majority of the participants were female (n = 87, 78.4%) and white (n = 67, 60.4%). The average age of the sample was approximately 44 years (standard deviation = 15.2 years; range, 22–82 years). At the time of completing the survey, 18 (16%) participants were 7 or less days since becoming symptomatic, 41 (37%) surveyed between 7 and 14 days, and 52 (47%) were ≥ 14 days since reporting symptoms indicative of RSV infection.

**Table 2:** Participant Characteristics and Descriptive Statistics of Additional Outcomes

Characteristic	Participants (N = 111)
Age, mean (SD), years	44.37 (15.2)
Median, minimum–maximum	41.0, 22–82
Sex, n (%)	
Male	24 (21.6)
Female	87 (78.4)
Race and ethnicity, n (%)	
White	67 (60.4)
African American	28 (25.2)
Asian	3 (2.7)
American Indian or Pacific Islander	0 (0.0)
Other	1 (0.9)
Hispanic	12 (10.8)
Symptoms experienced at day 1 due to RSV, n (%) (per screening)	
Cough	76 (68.5)
Cough with mucous	56 (50.5)
Stuffy nose	58 (52.3)
Runny nose	50 (45.0)
Sore throat	35 (31.5)
Body aches or pain	42 (37.8)
Shortness of breath	44 (39.6)
Fatigue	63 (56.8)
Sinus pain	28 (25.2)
Ear pain	20 (18.0)
Headache	49 (44.1)
Wheezing	32 (28.8)
Loss of appetite	38 (34.2)
PGIS at day 1, mean (SD) median	2.47 (0.8) 2.0
1 = None	9 (8.1)
2 = Mild	51 (45.9)
3 = Moderate	41 (36.9)
4 = Severe	10 (9.0)
PGIS at day 2, mean (SD), median	2.26 (0.7) 2.0
1 = None	12 (12.6)
2 = Mild	51 (53.7)
3 = Moderate	27 (28.4)
4 = Severe	5 (5.3)
Missing	16 (14.4)
PGIC at day 2, mean (SD) median	2.28 (0.9) 2.0
1 = Much better	20 (21.1)
2 = A little better	37 (38.9)
3 = No change	30 (31.6)
4 = A little worse	7 (7.4)
5 = Much worse	1 (1.1)
Missing	16 (14.4)
SDS Work/School, mean (SD) at day 1	4.95 (3.2)
Median, minimum–maximum	5.0, 0–10
SDS Family Life/Home Responsibilities, mean (SD) at day 1	5.08 (3.1)
Median, minimum–maximum	5.0, 0–10

PGIC = Patient Global Impression of Change; PGIS = Patient Global Impression of Severity; RSV = respiratory syncytial virus; SD = standard deviation; SDS = Sheehan Disability Scale

Because the web-based survey design did not allow for participants to skip questions, question-level missing data on the RSV-iiiQ could not be evaluated. On day 1, mean scores for individual items ranged from a low of 0.41 for Fever (Question 6) to a high of 1.64 for Cough (Question 1) (Additional File 1, Table S2) on the 0 (None) to 3 (Severe) response scale. All response options were endorsed for all questions; however, five questions exhibited skewness, with > 50% of the sample endorsing the lowest symptom score/impact (0) on both days: Fever (Question 6), Ear pain (Question 15), Hoarseness (Question 16), Dress yourself (Question 25), and Helpless (Question 27).

Some strong (r > 0.80) correlations among the Functional Impacts questions and the Emotional Impacts questions indicated possible redundancies in question content (data not shown). Despite the small sample size, all single-factor congeneric CFA models confirmed that each of the four scales was sufficiently unidimensional (Additional File 1, Table S2). Items within Systemic Symptoms, Functional Impacts, and Emotional Impacts loaded strongly on their respective factors (> 0.6) and satisfactory model fit was observed according to the standardized root mean square residuals (SRMRs), comparative fit indexes (CFIs), and the Tucker-Lewis Index (TLI). The fit of the single-factor model to the Respiratory Symptoms items was less satisfactory—the root mean square error of approximation (RMSEA) was somewhat large (RMSEA = 0.142) and the TLI was slightly low (TLI = 0.939 < 0.95), but all loadings were satisfactory (> 0.63). One source of this problematic fit was the high correlation between the residuals of two candidate items, Runny nose (Question 4) and Stuffy nose (Question 5). Alternative models were analyzed based on the provisional removal of two questions, Runny nose (Question 4) and Ear pain (Question 15). The resulting CFIs and TLIs exceeded 0.95, SRMRs were less than 0.06, and RMSEAs were mixed. Question-level test-retest reliabilities and construct validity results were adequate (all kappa coefficients ≥ 0.60 except 3—Runny nose, Fever, and Irritable; all item-total r values ≥ 0.42) (Additional File 1, Tables S2 and S3).

Scores based on the four hypothesized scales of the FluiiQ^™^ were calculated (see Additional File 1, Tables S1 and S2). Two questions, Runny nose (Question 4) and Ear pain (Question 15), had suboptimal psychometric properties that could potentially impair the performance of the RSV-iiiQ. Candidate scores were created with these two questions provisionally removed and analyses were performed to explore their impact on the RSV-iiiQ scores.

### Reliability

The test-retest reliabilities computed for the RSV-iiiQ scores based on no change in PGIC were mostly satisfactory (all ICCs except for the Emotional Impacts composite score were > 0.70) (Table 3), as were the internal consistency reliabilities (all alphas ≥ 0.87), which indicated that the proposed RSV-iiiQ scales were composed of items related to each other and the factor analyses supported the proposed scoring structure. The RSV-iiiQ scores with Runny nose and Ear pain provisionally removed were comparable in terms of test-retest and internal consistency reliabilities (data not shown) to the RSV-iiiQ scores that included Runny nose and Ear pain.

**Table 3:** RSV-iiiQ Score Descriptive Statistics, Reliability, and Construct Validity Correlations (Day 1, n = 111)

RSV-iiiQ Score	Mean (SD)	Test-retest ICC	Alpha Day 1, Day 2	Construct Validity Correlations (Day 1)										
				Sheehan Disability Scale				EQ-5D-5L						PGIS
				Work/ School	Family Life/ Home	Days Lost	Days Unpro- ductive	Mobility	Usual Activities	Self-care	Pain/ Discomfort	Anxiety/ Depres-sion	VAS	
Respiratory Symptoms	1.15 (0.7)	0.86	0.90, 0.90	0.65*	0.61*	0.34*	0.45*	0.41*	0.43*	0.37*	0.61*	0.26	−0.54*	0.78*
Systemic Symptoms	1.06 (0.7)	0.80	0.88, 0.87	0.61*	0.56*	0.36*	0.49*	0.43*	0.54*	0.51*	0.74*	0.32*	−0.56*	0.64*
Functional Impacts	0.99 (0.8)	0.80	0.94, 0.94	0.69*	0.67*	0.42*	0.48*	0.49*	0.61*	0.56*	0.61*	0.29	−0.57*	0.76*
Emotional Impacts	1.15 (0.9)	0.65	0.88, 0.91	0.66*	0.62*	0.36*	0.39*	0.38*	0.44*	0.40*	0.46*	0.45*	−0.51*	0.60*

* *P* < 0.01 for H_0_: ρ = 0

ICC = intraclass correlation coefficient; PGIS = Patient Global Impression of Severity; RSV-iiiQ = Respiratory Syncytial Virus Infection, Intensity and Impact Questionnaire; SD = standard deviation; VAS = visual analog scale

### Concurrent and known groups validity

All correlations between the RSV-iiiQ scores and the SDS scores were positive and moderate to strong (r ≥ 0.34), as expected (Table 3 and Additional File 1, Table S4). This indicated that participants with worse symptoms and impacts as measured by the RSV-iiiQ also demonstrated greater disability on the SDS. As predicted, the RSV-iiiQ Functional Impacts score was strongly correlated with the SDS Work/School score (r = 0.69) and the SDS Family Life/Home Responsibilities score (r = 0.67)—these were the strongest correlations between RSV-iiiQ scores and SDS scores. Positive correlations were expected and observed between the RSV-iiiQ and EQ-5D-5L domain scores (Table 3 and Additional File 1, Table S4), such that participants with worse RSV symptoms and impacts also reported poorer health status on the EQ-5D-5L domains. At day 1 and day 2, the Respiratory Symptoms and Systemic Symptoms scores were strongly correlated with the EQ-5D-5L Pain/discomfort domain, stronger than with other EQ-5D-5L domains. As hypothesized, the Functional Impacts score was strongly correlated with EQ-5D-5L Usual Activities at both time points (r = 0.61 and r = 0.60 at day 1 and day 2, respectively) and with EQ-5D-5L Self-care (r = 0.56 and r = 0.60 at day 1 and day 2, respectively). It was hypothesized that the Emotional Impacts score would be more strongly correlated with the EQ-5D-5L Anxiety/depression domain than with the other EQ-5D-5L domains, and although this was true at day 2 (r = 0.62), the day 1 correlation was 0.45, slightly smaller than the 0.46 correlation between Emotional Impacts and the EQ-5D-5L Pain/discomfort score; at both timepoints, the strongest correlations for the EQ-5D-5L Anxiety/depression domain were associated with the RSV-iiiQ Emotional Impacts score. As expected, all RSV-iiiQ scores were negatively correlated with the EQ-VAS score.

With respect to known groups validity, participants with an RSV diagnosis in the last 7 days had higher (more severe) average RSV-iiiQ scores compared with participants who had been diagnosed 14 or more days before; these mean differences were statistically significant for the Respiratory Symptoms and Functional Impacts scores (Table 4). Significantly worse (higher) mean scores on all RSV-iiiQ scores were reported by participants with Moderate or Severe PGIS ratings compared with participants who had PGIS ratings of None or Mild, indicating the RSV-iiiQ was capable of distinguishing between participants with None/Mild or Moderate/Severe PGIS ratings (Table 4). Scores on the SDS indicated that all mean differences between groups were in the hypothesized direction and statistically significant, demonstrating that study participants who reported experiencing moderate, marked, or extreme disruptions also experienced worse RSV symptoms and greater difficulties in functioning (Table 4). The results of additional analyses with Runny nose and Ear pain removed indicated that omitting these items did not substantively change any findings (data not shown).

**Table 4:** RSV-iiiQ Score Known Groups Validity (Day 1, n = 111)

RSV-iiiQ Score	Known Groups Mean (SD), *P* value of F test			
	PGIS Moderate or Severe (n = 51) vs. None or Mild (n = 60)	Time Since Diagnosis ≤ 7 days (n = 18) vs. ≥ 14 days (n = 52)	SDS Work/School > 5 (n = 34) vs. ≤ 5 (n = 45)	SDS Family Life/Home Responsibilities > 5 (n = 48) vs. ≤ 5 (n = 63)
Respiratory Symptoms	1.66 (0.7); 0.72 (0.5) *P* < 0.01	1.63 (0.9); 0.95 (0.6) *P* < 0.01	1.65 (0.6); 0.74 (0.5) *P* < 0.01	1.62 (0.6); 0.80 (0.6) *P* < 0.01
Systemic Symptoms	1.47 (0.7); 0.72 (0.5) *P* < 0.01	1.20 (0.8); 0.93 (0.7) *P* = 0.16	1.60 (0.6); 0.71 (0.6) *P* < 0.01	1.49 (0.6); 0.74 (0.6) *P* < 0.01
Functional Impacts	1.52 (0.7); 0.54 (0.6) *P* < 0.01	1.35 (1.0); 0.75 (0.7) *P* < 0.01	1.59 (0.7); 0.59 (0.6) *P* < 0.01	1.52 (0.7); 0.59 (0.6) *P* < 0.01
Emotional Impacts	1.57 (0.8); 0.79 (0.7) *P* < 0.01	1.17 (1.0); 1.05 (0.8) *P* = 0.61	1.76 (0.7); 0.86 (0.7) *P* < 0.01	1.64 (0.8); 0.77 (0.7) *P* < 0.01

PGIS = Patient Global Impression of Severity; RSV-iiiQ = Respiratory Syncytial Virus Infection, Intensity and Impact Questionnaire; SD = standard deviation; SDS = Sheehan Disability Scale

### Responsiveness

Although change was expected to be minimal from day 1 to day 2, all observed means of RSV-iiiQ scales showed statistically significant improvement (Table 5), indicating that RSV-iiiQ scores were sensitive to change in symptoms and impact over time; effect-size estimates were small, ranging from − 0.24 (Systemic Symptoms) to − 0.33 (Emotional Impacts). Moderate correlations (r = 0.31 to 0.36) were observed between all RSV-iiiQ change scores and PGIS change (except for Systemic Symptoms) (Table 5). Small correlations were observed between all RSV-iiiQ change scores and PGIC ratings. Responsiveness results were similar for the RSV-iiiQ scores with Runny nose and Ear pain omitted (data not shown).

**Table 5:** RSV-iiiQ Score Responsiveness: Effect-Size Estimates, Observed Score Changes, and Correlation Between Changes (n = 95)

RSV-iiiQ Score	Effect-Size Estimate	Score Change, Mean (SD), t (*P*)	Correlation Between Changes	
			PGIS Change	PGIC
Respiratory Symptoms	–0.26	–0.19 (0.4), 4.17 (< 0.01)	0.35*	0.22
Systemic Symptoms	–0.24	–0.17 (0.5), 3.54 (0.01)	0.22	0.26
Functional Impacts	–0.32	–0.26 (0.6), 4.55 (< 0.01)	0.31*	0.23
Emotional Impacts	–0.33	–0.29 (0.6), 4.49 (< 0.01)	0.36*	0.11

PGIC = Patient Global Impression of Change; PGIS = Patient Global Impression of Severity; RSV-iiiQ = Respiratory Syncytial Virus Infection, Intensity and Impact Questionnaire; SD = standard deviation

Note: Effect size is calculated as: (change from day 1 to day 2) ÷ (day 1 SD)

* *P* < 0.01 for H_0_: ρ = 0

## Discussion

This research describes the preliminary psychometric properties of the RSV-iiiQ, focusing on evidence of reliability and validity of data collected from a sample of people with acute RSV. The RSV-iiiQ allows adults to report a wide range of symptoms and impacts of RSV infection from the perspective of adults with acute RSV. The individual RSV-iiiQ questions performed well based on their observed distributional characteristics, test-retest reliability, construct validity, and known groups analyses. Importantly, the hypothesized single-factor structure, based on the subscales of the well-established FluiiQ™, was reproduced at the individual construct level, although some of the fit indexes for the Respiratory Symptom scale were unsatisfactory. In particular, RMSEA values are likely to be inflated for models with small sample sizes (e.g., N < 200) [28, 29]. However, the CFAs indicated that the questions worked well together and that the computation of RSV-iiiQ scores provided valuable summary information about the symptoms and impact of RSV. Our a prior hypothesized and observed associations between RSV-iiiQ scales and the PGIS, SDS, and EQ-5D-5L were largely consistent. Specifically, the moderate to strong correlations between the RSV-iiiQ and multiple measures of similar concepts provided evidence of content and construct validity. Known groups analyses provided support for the RSV-iiiQ’s discriminating ability. Importantly, while we expected small changes from day 1 to day 2, responsiveness analyses demonstrated observable differences, suggesting that this PRO may be sensitive to small differences in effectiveness between RSV-related symptoms and impact over time, potentially between intervention and control groups in clinical trials.

The potential usefulness of the RSV-iiiQ is further supported by the fact that it is based on the strong a priori model of the FluiiQ^™^ [9]. The RSV-iiiQ has been developed in accordance with FDA PRO guidance for medical product labeling that stipulates any PRO instrument referenced in product labeling must be developed with extensive input from patients and tested in the population involved in the clinical trials [21, 22]. The Respiratory Intensity and Impact Questionnaire (RiiQ™) is a different RSV-specific PRO based on the FluiiQ^™^ [30]. A third RSV-specific PRO, the Respiratory Infection-Patient Reported Outcomes (RI-PRO™), was adapted from the inFLUenza Patient-Reported Outcome instrument (FLU-PRO) [31]. However, a 2018 content validity assessment concluded that neither the RiiQ nor the RI-PRO are ideal for assessing symptoms of RSV [31].Although the RSV-iiiQ is primarily intended for use in clinical trials, it could be used in other contexts, such as epidemiological studies, where an assessment is needed of the symptoms and impact of RSV infection.

An important limitation of the present preliminary study is that a longitudinal assessment over the course of the disease, from the initial development of symptoms to full resolution, was not undertaken. We suggest that a longitudinal psychometric evaluation be conducted in the context of a future vaccine or treatment trial to evaluate the responsiveness of the RSV-iiiQ to change and to develop thresholds for meaningful within-patient change. Another limitation was that the present sample size was small, with 23% of the sample 60 years of age or older, including only 18 people proximal to symptom onset, although almost 50% of participants rated their symptoms as moderate or severe. Future research with a larger sample size using item response theory analyses could provide important information about the performance of the RSV-iiiQ items, especially regarding possible differential item functioning across groups of patients. While each of the RSV-iiiQ scales appear to have overall good properties, further psychometric work is necessary to demonstrate whether items cross load on non-target constructs. Although the present results indicate removal of the items Runny nose and Ear pain would not compromise the measurement properties of the RSV-iiiQ, all items are retained in the current version of the tool. The RSV-iiiQ composite scores should be revisited with further attention to the items Runny nose and Ear pain. We recommend further evaluation of the RSV-iiiQ in diverse settings to develop evidence about its measurement properties in different contexts and for different purposes [32].

## Conclusions

Our findings provide preliminary evidence that the RSV-iiiQ has satisfactory psychometric properties as a patient-reported measure of the symptoms and impact of RSV infection in adults with RSV. This study substantiates the findings of the qualitative research undertaken in the development of the RSV-iiiQ [10] and, taken together, both this study and the qualitative research support the conclusion that the RSV-iiiQ is likely to be useful for the assessment of the severity of symptoms and impact of acute RSV. However, further psychometric evaluation is necessary to evaluate the RSV-iiiQ responsiveness to change, to develop thresholds for meaningful within-patient change, and to demonstrate whether items cross load on non-target constructs. The findings also indicated two items, Runny nose and Ear pain, may be unnecessary and should be revisited using item response theory analysis with a larger sample size.

### Electronic supplementary material

Below is the link to the electronic supplementary material.

Supplementary Material 1

## Data Availability

Data are not available.

## Abbreviations

ANOVA: analysis of variance
CFA: confirmatory factor analysis
CFI: comparative fit index
EQ-5D-5L: 5-level EQ-5D
FDA: US Food and Drug Administration
FluiiQ^™^: Influenza Intensity and Impact Questionnaire
ICC: intraclass correlation coefficient
PGIC: Patient Global Impression of Change
PGIS: Patient Global Impression of Severity
PRO: patient-reported outcome
RMSEA: root mean square error of approximation
RSV: respiratory syncytial virus
RSV-iiiQ: Respiratory Syncytial Virus Infection, Intensity and Impact Questionnaire
SD: standard deviation
SDS: Sheehan Disability Scale
SRMR: standardized root mean square residual
TLI: Tucker-Lewis Index
US: United States
VAS: visual analog scale
