# Supplementary material for: Psychometric evaluation of the respiratory syncytial virus infection, intensity and impact questionnaire (RSV-iiiQ) in adults
Source: Health Qual Life Outcomes. 2024 Feb 20;22:19. doi: 10.1186/s12955-023-02174-2 (PMC10880342; doi:10.1186/s12955-023-02174-2)
Supplement: Supplementary file 1 — Supplementary Material 1 [file 12955_2023_2174_MOESM1_ESM.docx]

Table S1. Participant-Reported RSV Symptoms by Day

| Symptoms^a^ | Day 1 (N = 111) n (%) | Day 2 (n = 95) n (%) |
| --- | --- | --- |
| Cough | 76 (68.5) | 67 (70.5) |
| Cough with mucous | 56 (50.5) | 49 (51.6) |
| Stuffy nose | 58 (52.3) | 49 (51.6) |
| Runny nose | 50 (45.0) | 46 (48.4) |
| Sore throat | 35 (31.5) | 32 (33.7) |
| Body aches or pain | 42 (37.8) | 35 (36.8) |
| Shortness of breath | 44 (39.6) | 38 (40.0) |
| Fatigue | 63 (56.8) | 55 (57.9) |
| Sinus pain | 28 (25.2) | 25 (26.3) |
| Ear pain | 20 (18.0) | 18 (18.9) |
| Headache | 49 (44.1) | 41 (43.2) |
| Wheezing | 32 (28.8) | 28 (29.5) |
| Loss of appetite | 38 (34.2) | 35 (36.8) |

RSV = respiratory syncytial virus

^a^ Participants were instructed to check all symptoms that apply.

Table S2. Item-Level Descriptive Statistics and Single-Factor Confirmatory Factor Analysis at Day 1 (n = 111)

| RSV-iiiQ Question Content ^✝^ | Mean (SD) | % at Best Score, % at Worst Score | Single-factor CFA Loading (SE) | | | |
| --- | --- | --- | --- | --- | --- | --- |
|  |  |  | Respiratory Symptoms^a^ | Systemic Symptoms^b^ | Functional Impacts | Emotional Impacts |
| 1 Cough | 1.64 (0.9) | 7.2, 18.0 | 0.88* (0.03) | — | — | — |
| 2 Sore throat | 1.05 (1.0) | 35.1, 9.9 | 0.63* (0.06) | — | — | — |
| 3 Headache | 1.25 (1.1) | 31.5, 16.2 | — | 0.77* (0.05) | — | — |
| 4 Runny nose | 1.18 (0.9) | 27.9, 7.2 | 0.63* (0.06) | — | — | — |
| 5 Stuffy nose | 1.31 (1.0) | 26.1, 12.6 | 0.73* (0.05) | — | — | — |
| 6 Fever | 0.41 (0.8) | **73.0**, 2.7 | — | 0.82* (0.05) | — | — |
| 7 Body aches… | 1.10 (1.0) | 33.3, 9.0 | — | 0.82* (0.04) | — | — |
| 8 Fatigue… | 1.63 (1.0) | 14.4, 22.5 | — | 0.87* (0.03) | — | — |
| 9 Wheezing | 0.96 (1.1) | 45.9, 12.6 | 0.85* (0.03) | — | — | — |
| 10 Interrupted sleep | 1.60 (1.0) | 16.2, 23.4 | — | 0.76* (0.05) | — | — |
| 11 Loss of appetite | 1.01 (0.9) | 37.8, 6.3 | — | 0.64* (0.06) | — | — |
| 12 Cough with mucous… | 1.23 (1.0) | 26.1, 12.6 | 0.83* (0.04) | — | — | — |
| 13 Shortness of breath | 1.16 (1.0) | 32.4, 10.8 | 0.78* (0.05) | — | — | — |
| 14 Sinus pain | 0.94 (0.9) | 36.9, 2.7 | — | 0.79* (0.05) | — | — |
| 15 Ear pain | 0.56 (0.9) | **66.7**, 4.5 | — | 0.80* (0.05) | — | — |
| 16 Hoarseness | 0.80 (1.0) | **51.4**, 8.1 | 0.70* (0.06) | — | — | — |
| 17 Chest pain with coughing | 1.03 (1.0) | 36.0, 9.9 | 0.88* (0.03) | — | — | — |
| 18 Get out of bed | 0.95 (0.9) | 40.5, 4.5 | — | — | 0.92* (0.02) | — |
| 19 Prepare meals… | 0.87 (0.9) | 42.3, 6.3 | — | — | 0.82* (0.04) | — |
| 20 Perform usual… | 1.17 (0.9) | 27.9, 9.0 | — | — | 0.95* (0.02) | — |
| 21 Leave the home… | 1.13 (1.0) | 29.7, 10.8 | — | — | 0.94* (0.02) | — |
| 22 Concentrate on tasks | 1.10 (1.0) | 31.5, 9.0 | — | — | 0.91* (0.02) | — |
| 23 Take care of yourself | 0.83 (0.9) | 44.1, 5.4 | — | — | 0.91* (0.02) | — |
| 24 Walk up a flight of stairs | 1.28 (1.1) | 32.4, 19.8 | — | — | 0.84* (0.03) | — |
| 25 Dress yourself | 0.57 (0.9) | **64.0**, 4.5 | — | — | 0.84* (0.04) | — |
| 26 Irritable | 1.36 (1.0) | 24.3, 15.3 | — | — | — | 0.80* (0.04) |
| 27 Helpless | 0.76 (0.9) | **53.2**, 5.4 | — | — | — | 0.87* (0.04) |
| 28 Frustrated | 1.36 (1.0) | 25.2, 17.1 | — | — | — | 0.96* (0.03) |
| 29 Worried | 1.12 (1.0) | 33.3 12.6 | — | — | — | 0.82* (0.04) |
| **CFA Fit Indexes** |  |  | **Single-factor CFA Fit Indexes** | | | |
|  |  |  | **Respiratory Symptoms^a^** | **Systemic Symptoms^b^** | **Functional Impacts** | **Emotional Impacts** |
| RMSEA (optimal RMSEA < 0.05) |  |  | 0.142 | 0.113 | 0.120 | 0.102 |
| SRMR (optimal SRMR < 0.06) |  |  | 0.060 | 0.057 | 0.031 | 0.016 |
| CFI (optimal CFI > 0.95) |  |  | 0.954 | 0.974 | 0.993 | 0.998 |
| TLI (optimal TLI > 0.95) |  |  | 0.939 | 0.964 | 0.990 | 0.994 |

^✝^ Items truncated; full items available from Measured Solutions for Health P/L [info@measuredsolutions.com.au](mailto:info@measuredsolutions.com.au).

* *P* < 0.05 for H_0_: loading = 0.

CFA = confirmatory factor analysis; CFI = Comparative Fit Index; RMSEA = root mean square error of approximation RSV-iiiQ = Respiratory Syncytial Virus Infection, Intensity and Impact Questionnaire; SD = standard deviation; SE = standard error; SRMR = standardized root mean square residual; TLI = Tucker-Lewis Index.

^a^ Because of the strong correlation between Runny nose (Question 4) and Stuffy nose (Question 5), a reduced CFA model without Runny nose was evaluated; for this model, all loadings exceeded 0.63 at day 1, with improved model fit: RMSEA = 0.072, SRMR = 0.039, CFI = 0.991, TLI = 0.987.

^b^ Based on the question-level results for Ear pain (Question 15), a reduced CFA model without Ear pain was evaluated; for this model, all loadings exceeded 0.65 at day 1, with improved model fit: RMSEA = 0.128, SRMR = 0.055, CFI = 0.973, TLI = 0.959.

Table S3. Item-Total Correlation Coefficients at Day 1 (n = 111) and Day 2 (n = 95) and Test-retest Reliability from Day 1 to Day 2 (n = 30)

| RSV-iiiQ Question Content ^✝^ | Respiratory Symptoms (Day 1, Day 2) | Systemic Symptoms (Day 1, Day 2) | Functional Impacts  (Day 1, Day 2) | Emotional Impact  (Day 1, Day 2) | Test-retest Reliability Estimates (95% CI) |
| --- | --- | --- | --- | --- | --- |
|  |  |  |  |  | PGIC = No Change (3) |
| 1 Cough | 0.75, 0.69 | — | — | — | 0.74 (0.60-0.87) |
| 2 Sore throat | 0.55, 0.69 | — | — | — | 0.60 (0.36-0.83) |
| 3 Headache | — | 0.68, 0.67 | — | — | 0.74 (0.58-0.90) |
| 4 Runny nose | 0.50, 0.42 | — | — | — | 0.57 (0.27-0.88) |
| 5 Stuffy nose | 0.62, 0.56 | — | — | — | 0.68 (0.47-0.89) |
| 6 Fever | — | 0.59, 0.60 | — | — | 0.22 (−0.12 to 0.55) |
| 7 Body aches… | — | 0.72, 0.68 | — | — | 0.62 (0.40-0.85) |
| 8 Fatigue… | — | 0.74, 0.63 | — | — | 0.71 (0.54-0.87) |
| 9 Wheezing | 0.73, 0.76 | — | — | — | 0.82 (0.72-0.91) |
| 10 Interrupted sleep | — | 0.63, 0.62 | — | — | 0.75 (0.59-0.92) |
| 11 Loss of appetite | — | 0.56, 0.64 | — | — | 0.79 (0.64-0.94) |
| 12 Cough with mucous… | 0.72, 0.73 | — | — | — | 0.81 (0.67-0.95) |
| 13 Shortness of breath | 0.68, 0.67 | — | — | — | 0.69 (0.48-0.90) |
| 14 Sinus pain | — | 0.65, 0.66 | — | — | 0.79 (0.63-0.95) |
| 15 Ear pain | — | 0.62, 0.59 | — | — | 0.81 (0.68-0.94) |
| 16 Hoarseness | 0.62, 0.71 | — | — | — | 0.68 (0.41-0.95) |
| 17 Chest pain with coughing | 0.78, 0.73 | — | — | — | 0.74 (0.55-0.93) |
| 18 Get out of bed | — | — | 0.83, 0.84 | — | 0.63 (0.29-0.98) |
| 19 Prepare meals… | — | — | 0.74, 0.82 | — | 0.63 (0.32-0.94) |
| 20 Perform usual… | — | — | 0.86, 0.89 | — | 0.76 (0.58-0.93) |
| 21 Leave the home… | — | — | 0.86, 0.89 | — | 0.75 (0.55-0.95) |
| 22 Concentrate on tasks | — | — | 0.82, 0.67 | — | 0.71 (0.52-0.90) |
| 23 Take care of yourself | — | — | 0.82, 0.78 | — | 0.63 (0.37-0.88) |
| 24 Walk up a flight of stairs | — | — | 0.76, 0.76 | — | 0.75 (0.58-0.92) |
| 25 Dress yourself | — | — | 0.72, 0.77 | — | 0.73 (0.47-1.00) |
| 26 Irritable | — | — | — | 0.68, 0.72 | 0.53 (0.24-0.81) |
| 27 Helpless | — | — | — | 0.73, 0.80 | 0.67 (0.43-0.90) |
| 28 Frustrated | — | — | — | 0.83, 0.86 | 0.65 (0.41-0.90) |
| 29 Worried | — | — | — | 0.72, 0.81 | 0.60 (0.29-0.92) |

^✝^ All items truncated; full items available from Measured Solutions for Health P/L [info@measuredsolutions.com.au](mailto:info@measuredsolutions.com.au).

CI = confidence interval; PGIC = patient global impression of change; RSV-iiiQ = Respiratory Syncytial Virus Infection, Intensity and Impact Questionnaire.

Note: Test-retest reliabilities from day 1 to day 2 were computed using patients reporting “no change” on the PGIC (n = 30).

Table S4. Correlations between RSV-iiiQ scale score and the Sheehan Disability Scale and EQ-5D-5L

| RSV-iiiQ Score  correlations with SDS | SDS Work/School | | SDS Social Life/ Leisure Activities | | SDS Family Life/Home Responsibilities | | SDS Number of School/Workdays Lost | | SDS Number of Days Underproductive | |
| --- | --- | --- | --- | --- | --- | --- | --- | --- | --- | --- |
| **Day 1** |  | |  | |  | |  | |  | |
| Respiratory Symptoms | 0.65 | | 0.61 | | 0.61 | | **0.34** | | **0.45** | |
| Systemic Symptoms | 0.61 | | 0.54 | | 0.56 | | **0.36** | | **0.49** | |
| Functional Impacts | **0.69** | | 0.65 | | **0.67** | | **0.42** | | **0.48** | |
| Emotional Impacts | 0.66 | | 0.56 | | 0.62 | | **0.36** | | **0.39** | |
| RSV-iiiQ Score  correlations with EQ-5D-5L | EQ-5D-5L Mobility | EQ-5D-5L Usual Activities | | EQ-5D-5L Self-care | | EQ-5D-5L Pain/‌Discomfort | | EQ-5D-5L Anxiety/‌Depression | | EQ-VAS |
| **Day 1** |  |  | |  | |  | |  | |  |
| Respiratory Symptoms | 0.41 | 0.43 | | 0.37 | | **0.61** | | 0.26 | | **−0.54** |
| Systemic Symptoms | 0.43 | 0.54 | | 0.51 | | **0.74** | | 0.32 | | **−0.56** |
| Functional Impacts | 0.49 | **0.61** | | **0.56** | | 0.61 | | 0.29 | | **−0.57** |
| Emotional Impacts | 0.38 | 0.44 | | 0.40 | | 0.46 | | **0.45** | | **−0.51** |
| **Day 2** |  |  | |  | |  | |  | |  |
| Respiratory Symptoms | 0.38 | 0.51 | | 0.47 | | **0.53** | | 0.33 | | **−0.55** |
| Systemic Symptoms | 0.40 | 0.54 | | 0.52 | | **0.65** | | 0.39 | | **−0.65** |
| Functional Impacts | 0.51 | **0.60** | | **0.60** | | 0.57 | | 0.45 | | **−0.59** |
| Emotional Impacts | 0.31 | 0.44 | | 0.38 | | 0.51 | | **0.62** | | **−0.57** |

RSV-iiiQ = Respiratory Syncytial Virus Infection, Intensity and Impact Questionnaire; SDS = Sheehan Disability Scale; VAS = visual analog scale.

Note: Correlation coefficients in bold were hypothesized to be stronger than other correlation coefficients.
